# Multimodal Interventions to Improve the Management of Chronic Non-Malignant Pain in Primary Care Using Participatory Research

**DOI:** 10.3390/clinpract11030072

**Published:** 2021-08-26

**Authors:** Yolanda Morcillo-Muñoz, Maria Holgado Jiménez Castellano, Francisco Jose Díaz Exposito, Antonio Jose Sanchez-Guarnido, Miguel Gimenez Alcantara, Maria Isabel Baena-Parejo

**Affiliations:** 1 Primary Care RN, Andalusian Health Service District Campo de Gibraltar, 11207 Algeciras, Spain; 2 Primary Care RN, Andalusian Health Service District Cordoba, 14011 Córdoba, Spain; mariahjc14@gmail.com (M.H.J.C.); misabel.baena@juntadeandalucia.es (M.I.B.-P.); 3 Primary Care, Andalusian Health District South Cordoba, 14940 Córdoba, Spain; fradiex@gmail.com (F.J.D.E.); Miguelgimenezalcantara@gmail.com (M.G.A.); 4 Primary Care, Andalusian Health Service District Motril, 18600 Motril, Spain; antoniojose.sanchezguarnido@gmail.com

**Keywords:** chronic pain, exercise, cognitive therapy, self-management, medication therapy management, community-based participatory research, patient-centered care, primary health care

## Abstract

Background: The use of diverse therapies combined with a multidisciplinary approach and prevention initiatives for patients with chronic non-malignant pain (CNMP) can improve health and have a positive impact on psychotropic drug use and the self-management of pain. Purpose: This purpose of this study has been two-fold: to conduct a literature review with a view to selecting best evidence recommendations for CNMP and to prioritize self-care recommendations using a participatory methodology for the analysis and selection of interventions. Methods: A qualitative, descriptive, and documentary method based on participatory action research was used. Findings: Based on the study results, a multimodal psychosocial intervention program has been designed for CNMP that includes psychoeducational therapy, pharmacological therapy, physical exercise, and health assets. Discussion: The findings are consistent with previous studies underlining the need to invest in resources for the management of CNMP, including strategies for good differential diagnoses and pharmacological treatments combined with non-pharmacological treatments to confer greater well-being for people living with pain who want to participate in their own recovery.

## 1. Introduction

Chronic non-malignant pain (CNMP) is considered a public health problem due to its high prevalence among the population. The condition has a significant impact on the quality of life of patients and their families and is largely managed in the primary care setting [1]. Pain is influenced by the psychological, social, and biological factors of individuals. The International Association for the Study of Pain (IASP) describes pain as “an unpleasant sensory and emotional experience associated with actual or potential tissue damage, or described in terms of such damage”. This definition has been expanded to encompass both physiological, emotional, psychological, and social aspects of pain [2,3].

Under the maxim “constant pain needs constant control,” the nurse, social worker, and physician Cicely Saunders (1918–2005) introduced the concept of “total pain” as a multidimensional construct that includes the social environment and emotional processes of patients with chronic disease. In a similar line, expanded gate control theory of pain posited that sensory-discriminative, cognitive-evaluative, and motivational-affective factors affect how patients experience chronic pain. Pain catastrophizing and acceptance are two interrelated psychological constructs that also influence the perception of chronic pain. These constructs mediate the relationship between the unpleasant experience of pain and suffering [4] and are strongly associated with depression, anxiety, and poor prognosis [5]. Catastrophizing is characterized by “the tendency to magnify the threat value of pain and to feel helpless in the context of pain, and by a relative inability to inhibit pain-related thoughts in anticipation of, during or following a painful encounter” [6,7]. The Acceptance and Commitment Therapy (ACT) framework created by S. Hayes focuses on the acceptance of pain. ACT aims to promote committed action guided by values and help individuals take personal responsibility for pain control [8,9].

In the treatment of chronic pain, early detection is key as it can reduce catastrophizing thoughts and depression and improve patients’ ability to perform activities of daily living [10]. Given the psychological and social consequences of pain, more complex and comprehensive treatments that do not consist solely of taking psychotropic drugs are needed.

Among the factors associated with pain in women, highlighted the greater frequency of chronic diseases, especially musculoskeletal disorders, poorer functional status, higher levels of psychological stress, and less physical activity [11,12].

Combined therapy programs based on a multidimensional and multidisciplinary approach should be developed to improve the health of the general population and treat chronic pain.

These therapies should be coupled with prevention initiatives, information, physiotherapy, clinical guidelines, as well as psychotherapeutic and pharmacological treatment, rehabilitation, and psychoeducation for both patients and families. Such interventions, whose efficacy and effectiveness can be evaluated, can also have an impact on the use of psychotropic drugs and the self-management of pain [13,14].

The salutogenic model, which is based on resistance and resilience in response to everyday life situations, focuses on personal health assets to effectively manage conflictive situations [15]. Evidence suggests that the use of medications such as tricyclic antidepressants, serotonin reuptake inhibitors, and pregabalin [16], as well as cardiovascular exercise [15], patient education and counseling [17], ACT and mindfulness [18,19], self-management [20,21], a proper balance between activity and rest, stress management, emotion regulation, and appropriate physical exercise [22] are useful for managing chronic pain conditions.

As a conceptual framework to select the dimensions to be reviewed, the Patient-Reported Outcomes Measurement Information System (PROMIS) model was followed [23,24], based on the World Health Organization (WHO) definition of health. Three health areas were chosen: Physical Health, Mental Health and Social Health.

Study has a three-fold purpose: (i) to review the literature on CNMP and select the best evidence recommendations for chronic pain; (ii) to prioritize self-care recommendations using a participatory methodology involving the analysis and selection of interventions; and (iii) design a multimodal treatment program for people suffering from chronic pain.

These results are part of the development and evaluation of a multimodal intervention protocol through information technology in primary care, as part of a broader research program aiming to improve pain management. In this document we will focus on the second objective “to prioritize self-care recommendations, with participatory methodology”.

## 2. Methodology

A three-stage transformative, participatory, and qualitative approach based on participatory action research (PAR) was used, as a working methodology. A multimodal pain therapy (MPT) protocol was developed using the three-stage Emergence, Interaction framework [25].

Ethical aspects of this study were approved by the Cordoba Research Ethics Committee of the Andalusian Public Health System. Informed consent (S0452) was approved by the Cordoba Research Ethics Committee and completed by the participants.

### 2.1. Phase 1: Emergence

In the initial emergence phase, a multidisciplinary team was formed. The team comprised clinical and scientific experts from different fields of knowledge with more than 15 years of experience (5 family nurses, 3 family doctors, 1 social educator, 1 rehabilitation physician, 2 psychologists, 1 pharmacist, 1 public health physician, and 1 physiotherapist). All of them actively participate in the follow-up of patients with chronic pain in the primary sector of care. Participants in each category were recruited to represent diverse care settings from various provinces of Andalusia, Spain (Cádiz, Granada, Seville and Córdoba) and clinical settings (medicine, nursing, physiotherapy, pharmacy, public health and rehabilitation).

The problem was analyzed using a biopsychosocial approach by people with CNMP.

We conducted a systematic review, following SIGN guidelines. The literature was identified according to an explicit search strategy selected according to defined inclusion and exclusion criteria evaluated against consistent methodological standards. As a result, applying judgement allowed us to develop the synthesis of the main grounds of evidence and extract the recommendations in four dimensions: pharmacological therapy, psychoeducational therapy, physical exercise, and health assets, by consensus of the reviewers, which have been considered most applicable to clinical practice. All studies used for this review were ranked according taking into account their levels of evidence in accordance with the Scottish Intercollegiate Guidelines Network system [26].

A literature review of the databases PubMed, Campbell Library and Cochrane Library Plus as well as the internet search in order to find guidelines or other documents related to the objective of the study. The websites of various official organizations and institutions were consulted: World Health Organization (WHO) and Pan American Health Organization (PAHO), National Institute for Health and Care Excellence (NICE), Canadian Agency for Drugs and Technologies in Health (CADTH), CPG Infobase: Clinical Practice Guidelines, Scientific societies, was performed using the following key search terms: Chronic Pain (MeSH terms), Mind-Body Therapies (MeSH terms), Pain Management (MeSH terms), Acceptance and Commitment Therapy (MeSH terms), Multidisciplinary (MeSH terms), Pharmacological (MeSH terms) and Biopsychosocial Model (MeSH terms).

The search was limited to publications from Janury 2018 to October 2020. The indicator for locating the documents was that they had been published in the last 5–10 years. Priority was given to secondary research documents followed by primary research.

Manuscripts were retrieved and reviewed utilizing the following inclusion criteria: subjects with CNMP, defined as pain lasting for more than three months; adult participants (aged ≥ 18); experimental intervention aimed at reducing chronic pain that involves a physical component and a psychological and/or social component; multimodal intervention, pharmacological and non-pharmacological treatment; studies with more than 20 subjects in each treatment arm to reduce the bias that can occur in small samples; studies with statistical significance; mixed studies (qualitative and quantitative), randomized controlled trials, and non-randomized longitudinal studies (prospective and retrospective). Exclusion criteria included the following: patients with acute pain (duration < 3 months); patients with palliative, cancer-related or post-surgical pain; pediatric population; interventions involving surgery; studies with insufficient samples (<20 subjects), studies without abstracts and/or incomplete text, dissertations, editorials, letters, and non-English articles.

The recommendations were adapted to the level of understanding and cultural environment of the expert patients who participated in the selection and prioritization of the recommendations for their subsequent implementation and to evaluate their effectiveness.

### 2.2. Phase 2: Interaction

The nominal group comprised patients diagnosed with CNMP, caregivers, experiential experts, as well as the expert professionals who conducted the literature review, drafted the questionnaire, and participated as observers and moderators of the nominal group. They were recruited to participate in a 1-day workshop held in Cabra, Córdoba, Spain, on 14 February 2019.

To select and validate the priority recommendations for the target population, two actions were carried out as follows.

#### 2.2.1. Design of the Questionnaire

A 5-point Likert scale questionnaire containing closed and open questions was designed to measure the expert participants’ (patients and caregivers) degree of agreement with the items. The response anchors were: 5 (very high), 4 (high), 3 (moderate), 2 (low), and 1 (very low) (see Appendix A).

#### 2.2.2. Analysis of Content Validity

**Figure 1:**
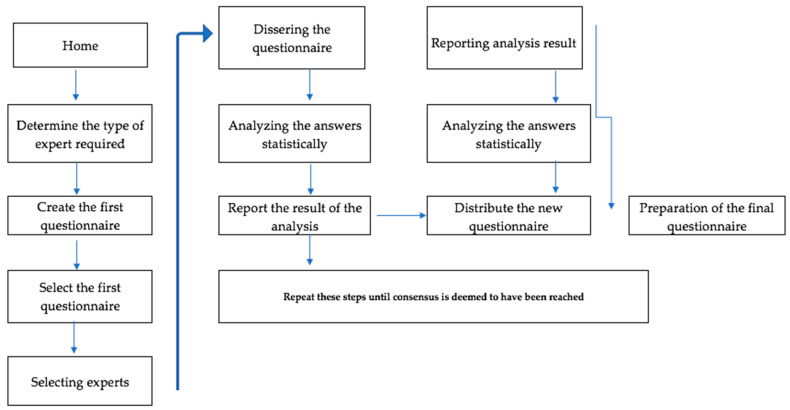
The Delphi Method procedure.

The content of the questionnaire was validated using a variant of the Delphi method. Specifically, we used the modified Delphi, what is known as the Estimate, Feedback, Talk, Estimate (EFTE), conversational, or face-to-face Delphi [27]. The Delphi Method consists of several stages: (i) analysis of the problem affecting the CNMP target population; (ii) selection of experts; (iii) presentation of the problem by means of a questionnaire to solicit responses; (iv) summary of the responses which are then transferred to a panel to identify the highest rated opinions, and (v) analysis of the information and consensus taking into account both divergent and convergent responses [28] (Figure 1).

The main objective of the dynamics was to validate and prioritize the recommendations obtained in the bibliographic review, grouped into the four dimensions identified in that review. We also sought to ensure that the final wording of the recommendations provided by the questionnaires was accessible and easy to understand. All this was materialized through group discussion by asking questions such as: “Can postural hygiene, relaxation and stretching exercises improve the health and quality of life of a person with chronic pain? Can managing emotions such as fear or sadness in the face of pain help me feel better? Can identifying the resources (or lack of them) that we have in our environment (family and community support networks, institutional, cultural or associative resources) help me to improve my health? To what extent does not abandoning the prescription of medications affect our health?

Through the completion of the questionnaires that included the recommendations which were the origin of these and other questions, and the feedback of the debate originated in the different rounds of the technique, the necessary degree of convergence of the individual estimates was reached, achieving consensus and stability in the panel’s responses, which guided the data analysis and decision making. The result was that a score was obtained for each recommendation within every dimension, which made it possible to estimate the consensus through the calculation of percentages, a measure agreed upon by the research group. It must be noted in this regard that there is not a single way of estimating consensus, according to the scientific literature on the subject.

Regarding the stability of the panel, the members of the research team who assumed the roles of moderator and observer determined that, once no significant variability in the opinions of the experts was achieved between successive rounds, the process was to be finished.

#### 2.2.3. Selection of Experts

The nominal group consists of a total of *n* = 36 participants from whom *n* = 8 were selected as principal investigators and co-researchers for their experience and management in the nominal group and *n* = 28 expert patients, including *n* = 20 women and *n* = 8 men who are familiar with and manage their CNMP process, who were intentionally selected.

To obtain heterogeneous responses, we partnered with the Local Health Action Network, Fibromyalgia Association, Socio-educational Groups, Trainers of The Patient School of the southern health area of Córdoba, to recruit patients and family members.

Applying a theory called “Belbin’s team roles”, the team meets the ideal profiles for the development of this research work [29].

Thus, we can find that in homogeneous groups, as in our case, the approximate size of a Delphi panel is generally less than 50; in heterogeneous groups, it can be hundreds.

The inclusion criteria for patients in this study were: individuals with chronic musculoskeletal pain, patients ≥ 18 years of age with pain of any location, with a duration of ≥3 months, an intensity of ≥4 on the Visual Numerical Scale (VNS), and with one of the following characteristics: continuous pain, intermittent pain ≥ 5 days a week.

All participants were asked to sign an informed consent form.

#### 2.2.4. Procedure

The participants in the nominal group were randomly divided into four smaller groups of 7 participants each in different rooms. Each group was led by two professional experts (literature reviewers and those who designed the questionnaire). One of the experts acted as the moderator and the other as the observer. The moderator was responsible for informing the participants about the session and handing out the questionnaires with the recommendations selected for each dimension (psychoeducational, pharmacological, health assets, and physical exercise) so that each participant could analyze and present their ideas. The observer was responsible for taking observational notes on the behavior, interactions, and verbalizations of the group members.

First, a small group discussion was held to reach consensus on the proposed priorities for each dimension, followed by a discussion with all participants. The group was asked to rate each idea using a previously defined 5-point quantitative scale in which the highest value was assigned to the approach considered most important. The scores obtained for each of the ideas were then transcribed. Once the scores were tallied, they were written down and a discussion was held on the points for which agreement had not been reached. The proposals were put to a new vote and the participants voted again for the factors they considered most important. The votes were then counted by factor, the responses were ordered from the highest to the lowest numbers of votes, and the results were read out loud to the participants. Given the substantial number of recommendations selected by the participants, the group agreed that there should be no more than 8 recommendations in each of the 4 dimensions of the approach to CNMP. Finally, specific recommendations were made for physical exercise, psychoeducation, health assets, and pharmacological therapy for the treatment of CNMP.

## 3. Results

A total of 225 citations were identified during the search, which after a first reading were excluded because they did not meet the objective of the study. After a second review, 42 citations were included. Eight documents were located in other databases, of which five were selected. The final review included 14 studies, of which six were systematic reviews, five were narrative reviews and three were clinical guidelines.

Table 1 displays descriptions of the included studies on therapy (method and type of Intervention).

In order to analyze the results, the statistical procedures were carried out according to the nature of the research conducted. With the total score obtained for each recommendation, as well as its maximum and minimum scores, the median was calculated, given the type of scale used (Likert) and the average to prioritize among the recommendations with the same median of the same dimension.

As the questionnaires included open-ended questions, as well as the account provided by the observers, a critical analysis of the discourse was carried out, which provided a battery of suggested recommendations which could be included in the multimodal protocol and that had not previously been reflected in the questionnaire provided.

Another result obtained thanks to the interaction between the members of the panel of experts was to adapt the wording of the recommendations or make it as adequate as possible to a clear, concise, accessible and understandable language for the target population.

Table 2 displays the frequency of the expert patients’ opinions on the final questionnaire in response to the general question: “To what degree can the following recommendations improve your health?”. As can be seen in the table, all the recommendations obtained high scores for their incorporation into the multimodal intervention program. Specifically, pharmacological therapy showed the highest frequency (87%), followed by health assets (85%), psychoeducational therapy (84%), and physical exercise (83%).

Table 3 shows the scores for the closed questions on recommendations for physical exercise. The priority recommendations were “Perform exercises and activities that are enjoyable and can be incorporated into daily routines” (R6), “Do exercise with the support of professional trainers during physical exercise programs to achieve long-term exercise goals and adherence” (R7), and “Perform postural hygiene, relaxation, and stretching exercises, as appropriate” (R1).

As regards psychoeducational therapy (Table 4), the priority recommendations were “Being able to keep on doing activities that are important in my daily life (family, work, leisure, friendships, etc.) helps me feel better” (R4) and “Relaxation or meditation helps me distance myself from pain and reduce my suffering” (R3).

The experts’ priority recommendations regarding health assets are shown in Table 5. Two main recommendations were selected: “Focusing on what makes us feel better increases the control we have over our health” (R5) and “Strengthen and enhance your personal assets, i.e., your virtues and skills (coping, social skills, commitment to learning, manual skills)” (R2).

Table 6 shows the selected pharmacological recommendations. These included “Make sure only one doctor prescribes pain relief medications. If another one changes the medicine, the two doctors should discuss the treatment together” (R4); “Make sure you do not run out of pain relief medications. Remember that prescriptions are needed to obtain opiate painkillers. Doctors cannot order them and pharmacies do not always have them in stock. It may take a few days for you to get the medicine, so include delays in planning” (R5); “Do not use medications that have expired or were used to treat other health problems. Medications that were effective in the past may not be suitable now” (R7).

In what follows, we present the responses of the nominal group to the open-ended question in the section titled “Other recommendations (briefly describe the recommendation and indicate the degree to which you think it can improve your health)”. The participants were asked to provide recommendations for aspects not reflected in the questionnaire but that could be included in the multimodal protocol. As regards exercise, the expert patients indicated that walking and receiving massages can improve their health. In relation to the psychoeducational component, they pointed to the need to combine therapies and work with professionals from different specialties. For the health assets component, they recommended engaging in cooperative community activities that provide CNMP patients with the opportunity to share their experiences, while in the pharmacological section, the participants referred to their lack of confidence in generic drugs and the importance of shared decision making and drug safety.

Table 7 shows the results of the contents of the multimodal intervention program based on the recommendations for the four thematic areas. We considered three elements: design, contents, and structure of the guide, which includes automatic monitoring, skills training, social support, education, goal setting, and achieving the four components of exercise, psychological well-being, pharmacological therapy, and health assets.

## 4. Discussion

This study has prioritized evidence-based interventions for the management of CNMP with a participatory methodology, a public health problem that entails a significant cost burden not only for patients and their families, but also for the health system as a whole as they frequently resort to primary healthcare and emergency services and may even require hospitalization.

To gather the opinions and identify the demands of the CNMP patients, a participatory method was used. The aim was to place these patients at the center of their own transformation and empower them, as well as to give them the opportunity to create support networks and strategic connections among the community stakeholders.

As the results have shown, the expert patients reached a consensus regarding the prioritization and choice of recommendations as a strategy for implementing the multimodal CNMP treatment program. Through collaboration between professionals and expert patients, as well as the application of diverse treatments that provide health benefits, practical solutions to complex problems have been found. The priority recommendations of the expert patients are in line with the reviewed literature, which has shown that multimodal treatments are preferable to single treatments [26]. Other authors have also pointed out the suitability of interdisciplinary care for people suffering from chronic pain. Such care should be based on a biopsychosocial approach, with individualized therapeutic plans and a comprehensive approach to health that not only includes treatment, but also diagnosis and continuous evaluation [39]. In this regard, engaging patients in decision making is especially useful to reach a joint resolution, which must make intellectual, emotional, and practical sense [40].

In the primary care setting, the prescription of medications, including opioids [38], is the primary treatment for pain patients with complex medical histories who often have multiple overlapping causes [41]. To ensure the safe use of opioids, patients must be informed of the benefits, risks, and adverse effects [37]. A wide range of medications are available for pain management, including non-opioid analgesics (non-steroidal anti-inflammatory drugs, COX-2 inhibitors, acetaminophen) and adjuvant medications that are used to relieve pain such as anticonvulsants, serotonin reuptake inhibitors, and muscle relaxants [42]. To prevent addiction, adverse effects, and increasing costs of pain management, it is important to assess the harms and benefits of medications and understand patients before continuing treatment [43]. 

Non-pharmacological therapy and non-opioid pharmacological therapy are preferred for chronic pain. If opioid therapy is considered, the expected benefits/risks should be assessed. If opioids are used, they should be combined with non-pharmacological therapy and non-opioid pharmacological therapy, as appropriate [38]. Moreover, as an integral part of pain management, multimodal and interprofessional programs geared toward chronic pain self-care have become necessary [21]. Indeed, self-care can have highly positive effects on pain patients as they believe in their own ability to control their pain. Considering the opinions of the patients puts the emphasis on the importance of shared decision making and drug safety.

Self-care programs under the guidance of health professionals may also influence health outcomes, such as preventing catastrophizing thoughts that modulate pain, such as the belief that pain cannot be controlled and exaggerating the threat. In this regard, self-care can improve patients’ capacity for self-management as they actively participate in their treatment. As Moore et al. [44] suggested, it is important to actively engage patients in problem solving, decision making, the utilization of healthcare resources, and encourage them to take action to manage their pain.

The findings of this work are consistent with previous studies on the need to invest in resources for pain management that include strategies for good differential diagnoses and pharmacological treatments combined with non-pharmacological treatments, as they can be beneficial for patients not only in terms of improving pain, but also in reducing anxiety and depression, facilitating rest, improved well-being, and the desire to participate in their own recovery.

For people with chronic pain, multiple non-pharmacological therapies are also available, such as physical activity to improve coordination, reduce pain, and improve mood.

According to our results, the R4 statement, “it is important to be able to continue doing activities that are in my daily life (family, work, leisure, friends, etc.) and help me feel better” is consistent with the evidence found on ACT [19]. 

Thus, in a clinical trial conducted in Spain on the cost-utility of ACT and the clinical evolution of CNMP, the subjects employed a variety of methods to promote psychological flexibility, such as exposure-based techniques, metaphors, mindfulness, and training activities compared to pharmacological treatment. The results showed that ACT is a cost-effective treatment compared to recommended medications [45].

Our results also found that the statement related to relaxation or meditation as a means of distancing oneself from pain and reducing suffering is in line with the results of studies on cognitive therapy and mindfulness-based stress reduction (MBSR), which comprises a state of awareness that has been characterized as non-elaborative, non-judgmental present-centered awareness and a form of acceptance and trust in one’s own experience; these studies have also reported reduced pain and physical well-being [46].

Expert patients prioritized, regarding physical activity, evidence-based recommendations to prescribe different types of exercises for the management of CNMP. Although a wide variety of exercises are recommended, it is important to take into account factors such as the intensity, duration and amount of the exercise, as well as prescribe individualized exercise to improve adherence and achieve good outcomes [31,47]. The proposed exercises and activities range from empowerment, stretching and relaxation, to walking or low-intensity activities of daily living that can help patients acquire habits and learn about alternatives to improve their daily lives [30,48].

The evidence presented in the narrative review of Pumar-Méndez et al. [33] suggests that new strategies for managing chronic illness such as self-care are more effective when approached at different levels (individual, community, organizational and systemic levels). According to qualitative research findings, patients who engage in self-care tasks require psychosocial and relational support from relatives and healthcare professionals. According to the key informants that participated in the study, it is important to empower patients so that they can better self-manage their process and make the best treatment decisions, as well as to involve patient associations that can influence the health policies and practices of healthcare professionals. The informants also highlighted the paternalistic role of healthcare professionals. Although they noted that attitudes towards patient autonomy and self-care are improving, there is still a long way to go due to the overexposure of health professionals to medicalized paradigms and barriers for implementing patient-centered care due to time constraints, training, resources, incentives and autonomy.

The expert patient opinions regarding the prioritized recommendations on health assets highlight the benefits of the asset approach for the population and the effective practices that serve as a strengthening of its capacities that can serve for a better autonomy. The importance of identifying health assets (family networks, friendship, community cohesion) as a strategy lies in the fact that they can be a resilience factor for disease exposure and furthermore can be a positive health entity in their own right, with a focus on quality of life and well-being, necessary to promote physical, mental and social health. Another inherent idea is that of asset mapping, whose technique is based on the search for community capacities, using individual and organizational resources in order to achieve better health management [32,34,36].

The health assets model provides a health perspective that encourages people to reorient their gaze to the context and focus on what improves health and well-being, strengthening their decisions about the root causes of their health problems. The recommendations of this dimension, incorporated into the multimodal intervention program, favor this line of action [35,49,50].

To ensure patient satisfaction and lower risks, it is essential that health professionals and researchers work together to translate clinical outcomes into value-based healthcare. Moreover, care must be personalized, and each patient’s personal situation must be taken into account to ensure that the health professional and the patient address health problems jointly from both a practical and an emotional perspective.

Our research on chronic pain has as its main strength the use of an active participatory methodology to prioritize the recommendations of the best evidence found in the review of the scientific literature. However, our manuscript also has some limitations, such as not developing a comprehensive systematic review of the recommendations. Therefore, here we aim to show the search strategy and the most important findings, with the review being itself separately dealt with and published due to its remarkable length.

This article also does not focus on the applications and concrete interventions to be developed from these recommendations, but the research group of this paper is working on the development of intervention programs and their evaluation, including a clinical trial to evaluate the effectiveness of a mobile application based on these recommendations.

The methodology used has the advantage that the opinion and experience of a nominal group provides higher quality compared to that of a single expert, but it must be taken into account that the bias of the participants due to their cultural background, age and gender, may have an impact in some cases. For this reason, the choice of experts was careful, inviting groups with a certain homogeneity. The use of the modified Delphi, called conversational or “face-to-face” Delphi, allows each participant to express him/herself freely, but also implies the possibility that some may be tempted to join the score closest to that of the total group, without their own argument. One of the limitations of this technique is that the results depend on the accuracy of the questions. That is, there must not be any doubt about their contents because the interpretation must be unique and real.

The length and wording of the sentences must be estimated and assessed. The greater the number of words in a question, the greater the possibility of error in its interpretation. To mitigate this situation, the methodology used incorporated two members of the research team, one as a moderator/informant and the other as an observer, thus minimizing the aforementioned risk.

The study focuses on those treatments for chronic pain available in the primary care setting. The interventions by invasive technique, are performed in the hospital setting in the pain units and will be derived if after 6 months of treatment and adequate follow-up with a comprehensive treatment plan the intensity of the pain and/or functionality have not reached the objectives, then it should be considered as a chronic pain picture of difficult control. In our health system, for the proper management and treatment of Chronic Pain, it has been proposed to integrate care into multidisciplinary teams (professions and specialties) within a pain care network; it would be an integrated network of services that guarantee a continuous care that would include, in addition to a Hospital Pain Treatment Unit, other specialized units of the same hospital or a different one, of different complexity, as well as other health resources such as Primary Care, social and socio-sanitary services. For its development, resources are needed, as much as protocols that guarantee the continuity of care and clinical integration of care teams along with information systems.

The results of this study are consistent with the reviewed literature and the expert patients’ knowledge and ideas for the treatment of CNMP.

Based on the results of the first phase of the study, a multimodal therapy application was designed for use in mobile devices. A clinical trial was then carried out to evaluate the effectiveness of a personalized intervention program using the mobile device to improve self-management and self-care at home. The aim was to assess the effectiveness of the application in reducing catastrophizing behavior, acceptance, emotional distress, and symptoms in people with CNMP.

## 5. Conclusions

Finally, we can say that chronic pain is one of the major problems that public health systems have to face and that the response that these systems give is very important and should be considered from a multidisciplinary and person-centered approach. Thus, in addition to the necessary individualized pharmacological treatment, other interventions focused on the psychological aspects of pain are necessary, favoring a response of acceptance and centered on values, but also interventions that include recommendations on physical activity and the strengthening of health assets.

A central element of our reflection of the participatory action research strategy is the path that we must walk together, both health professionals as well as patients and health institutions are to be an active part of the process, including in the clinical pathsheet shared decision making, respect, humanization of care and personalization of treatments that best suit the needs of the patient, in order to improve quality of life and self-management of chronic pain.

The results of this study may contribute to the implementation of new policies for the management of people with chronic pain in primary care, being of interest not only at the community level but also for health professionals who care for these patients. High quality clinical practice guidelines and clinical trials have been found in relation to pharmacological and non-pharmacological treatments for the coordinated management of patients with chronic pain. However, more research and resources are needed in primary care to provide more efficient and effective care with the best available evidence.

## Figures and Tables

**Table 1:** Description of Included Studies on Therapy (Method, Type of Intervention).

Therapy	Method	Type of Intervention
Physical Exercise	Clinical Practice Guideline	Ability to perform activities of daily living, “The recommended quality and quality of exercise for developing and maintaining cardiorespiratory and muscular fitness and flexibility in healthy adults” (Garber et al., 2011b, [30])
	Systematic review	Any intervention performed in primary, outpatient, or community care; self-care program; exercise combined with adherence component (Aitken et al., 2014, [31])
	Systematic review	Physical activity interventions, resistance, aerobic and combined programs; structured exercise (class, gym, at home) and unstructured exercise (activities of daily living) (Geneen et al., 2017a), (Geneen et al., 2017b, [22])
Psicoeducation	Systematic review	Intervention primarily with psychological content; cognitive behavioral therapy and multimodal intervention (physical and psychological content) (Schütze et al., 2018, [7])
	Systematic review	Intervention primarily with psychological content (Martinez-Calderon et al., 2018, [10])
	Systematic review	Acceptance and commitment and mindfulness intervention. Relaxation therapy(Veehof et al., 2016, [19])
Health assets	Narrative review	Community participation, that are effective in improving the population’s health. Peer-managed interventions (NICE 2016, [32])
	Narrative review	Interviews with key informants. Self-management capacity of patients with chronic diseases (Pumar-Méndez et al., 2017, [33])
	Narrative review	Cognitive component, instrumental component, motivational component (Rivera de los Santos et al., 2011, [34])
	Narrative review	Health assets, positive health (Cofiño et al., 2016, [35])
	Narrative review	Health assets, positive health (Morgan et al., 2010, [36])
Pharmacological	Clinical practice guideline	Multidisciplinary treatment; psychological, physical, and pharmacological interventions (Scottish Intercollegiate Guidelines Network 2013, [26])
	Clinical practice guideline	Patient participation, create panels and committees, and manage conflicts of interests. Recommend safe use of opioids and avoid adverse effects from long-term use (Busse et al., 2017, [37])
	Clinical practice guideline	Data from experts, peer reviewers, and a government-sanctioned advisory committee. Improve communication between healthcare professionals and patients about the risks and benefits of opioid therapy for chronic pain (Dowell et al., 2016, [38])

**Table 2:** Frequencies of expert patient opinions on the final questionnaire.

Likelihood of Ocurrence	Physical Exercise		Psychoeducation		Health Assets		Pharmacological	
	Frequency	%	Frequency	%	Frequency	%	Frequency	%
High	139	83	88	84	100	85	149	87
Moderate	18	11	15	14	15	13	15	9
Low	11	7	2	2	2	2	7	4
Overall	168	100	105	100	117	100	171	100

**Table 3:** Degree to which physical exercise recommendations can improve health.

Chronic Pain Patients (P1 to P20)	P1	P2	P3	P4	P5	P6	P7	P8	P9	P10	P11	P12	P13	P14	P15	P16	P17	P18	P19	P20	Frequency
Items Questionnaire (R1 to R9)	* Scale																				
R1 Perform postural hygiene, relaxation, and stretching exercises, as appropriate.	4	3	3	4	5	5	5	1	5	4	4	4	5	4	4	5	4	5	5	5	84
R2 Perform regular (150 min per week) and moderate aerobic physical exercise (walking, swimming, cycling) 3 days per week or 30 minutes per day 5 days a week.	4	4	4	4	3	4	1	3	5	3	5	4	5	4	5	5	5	5	5	5	83
R3 Do muscle resistance training 2 or 3 days a week, alternating between 8–12 repetitions. Rest muscle groups for at least 48 h between sessions. Older adults should do light or very light intensity exercises depending on their physical condition.	4	4	2	2	5	4	2	0	4	3	4	4	4	3	5	4	4	4	5	5	72
R4 Improve flexibility by stretching 2–3 times per week for 10–30 s and repeat 2 to 4 times. Stretching should be done after warming up muscles and after aerobic exercise to avoid injury.	4	4	3	4	4	5	5	5	4	4	4	4	5	4	4	5	5	0	4	4	81
R5 Perform exercises to increase agility, balance, coordination, and gait. Do multi-component activities, such as tai chi and yoga.	4	5	4	4	5	4	4	0	4	3	4	5	5	3	3	5	5	5	5	5	82
R6 Perform exercises and activities that are enjoyable and can be incorporated into daily routines.	5	4	4	4	5	5	5	3	5	5	4	5	5	4	4	5	5	5	5	5	92
R7 Do exercise with the support of professional trainers during physical exercise programs to achieve long-term exercise goals and adherence.	4	5	4	4	5	5	5	0	4	5	4	5	4	4	5	5	4	5	5	5	87
R8 Do exercise in refresher sessions with supplementary audiotape or videotape exercise material.	3	3	3	2	2	2	2	0	3	2	5	4	3	4	4	4	5	4	5	5	65
R9 Other physical exercise recommendations (briefly describe the recommendation in the box below and indicate the degree to which you think it can improve your health).	0	4	5	4	5	0	0	0	5	5	4	0	5	0	0	5	0	0	0	0	42

* Hight (5 to 4); Moderate (3 to 2); Low (1 to 0).

**Table 4:** Degree to which psychoeducational recommendations can improve health.

Chronic Pain Patients (P1 to P19)	P1	P2	P3	P4	P5	P6	P7	P8	P9	P10	P11	P12	P13	P14	P15	P16	P17	P18	P19	Frequency
Items Questionnaire (R1 to R6)	* Scale																			
R1 Certain emotions such as fear and sadness make me suffer more pain. Managing these emotions would help me feel better.	5	4	4	3	5	4	4	5	5	5	5	3	5	3	5	4	4	2	4	79
R2 Catastrophizing thoughts about pain make me feel worse; some acceptance of pain would help me suffer less.	4	3	5	4	5	4	4	5	5	5	5	4	5	5	5	3	3	1	4	79
R3 Relaxation or meditation helps me distance myself from pain and reduce my suffering.	4	5	4	4	4	3	4	5	5	4	5	4	3	5	5	4	4	5	4	81
R4 Being able to keep on doing activities that are important in my daily life (family, work, leisure, friendships, etc.) helps me feel better.	5	5	5	4	4	5	4	5	5	5	5	3	5	5	5	5	4	5	4	88
R5 Psychological therapy can help me feel better and reduce my suffering.	4	4	3	3	4	5	4	5	5	5	5	3	5	3	5	3	4	5	4	79
R6 Other psychoeducational recommendations (briefly describe the recommendation in the box below and indicate the degree to which you think it can improve your health).	4	NA	NA	4	5	NA	NA	5	NA	4	5	5	NA	NA	NA	NA	5	4	4	45

* Hight (5 to 4); Moderate (3 to 2); Low (1 to 0); Do not answer (NA).

**Table 5:** Degree to which health assets recommendations can improve health.

Chronic Pain Patients (P1 to P20)	P1	P2	P3	P4	P5	P6	P7	P8	P9	P10	P11	P12	P13	P14	P15	P16	P17	P18	P19	P20	Frequency
Items Questionnaire (R1 to R6)	* Scale																				
R1 Participating in group meetings and self-know-how spaces.	5	5	0	5	3	4	4	4	5	5	5	5	5	4	4	4	4	5	4	4	84
R2 Strengthen and enhance your personal assets, i.e., your virtues and skills (coping, social skills, commitment to learning, manual skills).	3	4	4	5	4	4	5	4	5	5	5	5	5	5	5	4	4	4	4	4	88
R3 Identify the resources (or resource shortfalls) in your environment (e.g., family and community support networks, institutional and cultural resources, associations).	5	5	1	5	3	4	4	4	5	5	5	5	1	4	5	3	5	2	5	5	81
R4 Reflect on the reasons that help you understand why certain situations occur.	2	4	0	5	3	4	5	4	4	5	4	4	1	4	4	3	4	4	3	3	70
R5 Focusing on what makes us feel better increases the control we have over our health.	4	5	3	5	5	4	5	5	5	5	5	5	5	4	4	4	4	4	4	4	89
R6 Other health assets recommendations (briefly describe the recommendation in the box below and indicate the degree to which you think it can improve your health).	5	5	NA	5	4	4	5	4	NA	5	NA	5	5	5	4	NA	NA	NA	NA	NA	56

* Hight (5 to 4); Moderate (3 to 2); Low (1 to 0); Do not answer (NA).

**Table 6:** Degree to which pharmacological recommendations can improve health.

Chronic Pain Patients (P1 to P20)	P1	P2	P3	P4	P5	P6	P7	P8	P9	P10	P11	P12	P13	P14	P15	P16	P17	P18	P19	P20	Frequency
Items Questionnaire (R1 to R10)	* Scale																				
R1 Take pain relief medication on a regular schedule to help manage pain. Take your medication just as prescribed, even when you don’t feel pain.	5	4	4	3	3	4	3	3	3	3	4	2	5	4	4	3	1	4	5	NA	67
R2 Don’t miss doses of the prescribed medication. The more pain you feel, the harder it will be to manage.	4	4	5	4	5	4	4	5	3	4	5	5	4	4	4	3	4	4	5	4	84
R3 If the pain is sudden, use fast-acting medication as prescribed by your doctor. Don’t wait until the pain intensifies; if you do, it can be harder to control.	3	4	3	4	4	4	2	4	4	3	3	4	4	4	4	2	3	4	3	3	69
R4 Make sure only one doctor prescribes pain relief medications. If another one changes the medicine, the two doctors should discuss the treatment together.	5	4	5	5	4	5	5	5	4	4	5	5	4	5	4	4	4	5	5	5	92
R5 Make sure you don’t run out of pain relief medications. Remember that prescriptions are needed to obtain opiate painkillers. Doctors cannot order them and pharmacies do not always have them in stock. It may take a few days for you to get the medicine, so include delays in planning.	5	4	5	4	4	5	5	5	4	4	5	5	4	4	5	4	4	5	5	5	91
R6 Never take medications that were prescribed to others. Medications that were effective for a friend or family member may not be appropriate for your situation.	4	5	5	4	4	5	5	5	4	5	4	5	4	4	5	4	4	5	5	4	90
R7 Don’t use medications that have expired or were used to treat other health problems. Medications that were effective in the past may not be suitable now.	5	4	5	4	4	5	5	5	4	4	5	5	4	4	5	4	4	5	5	5	91
R8 Painkillers affect different people in different ways. A very small dose may be effective for you, while others may require a much higher dose to relieve pain.	5	4	3	3	3	4	3	3	3	3	4	2	5	4	4	3	1	4	5	2	68
R9 Remember that your pain management plan may be modified from time to time.	4	4	5	4	5	4	4	5	3	4	4	5	4	4	4	3	4	4	5	4	83
R10 Other pharmacological recommendations (briefly describe the recommendation in the box below and indicate the degree to which you think it can improve your health).	NA	3	4	4	NA	NA	3	NA	4	NA	3	NA	NA	4	NA	4	NA	3	NA	NA	32

* Hight (5 to 4); Moderate (3 to 2); Low (1 to 0); Do not answer (NA).

**Table 7:** Multimodal intervention: physical exercise, pharmacological treatment, Psychoeducation, health assets.

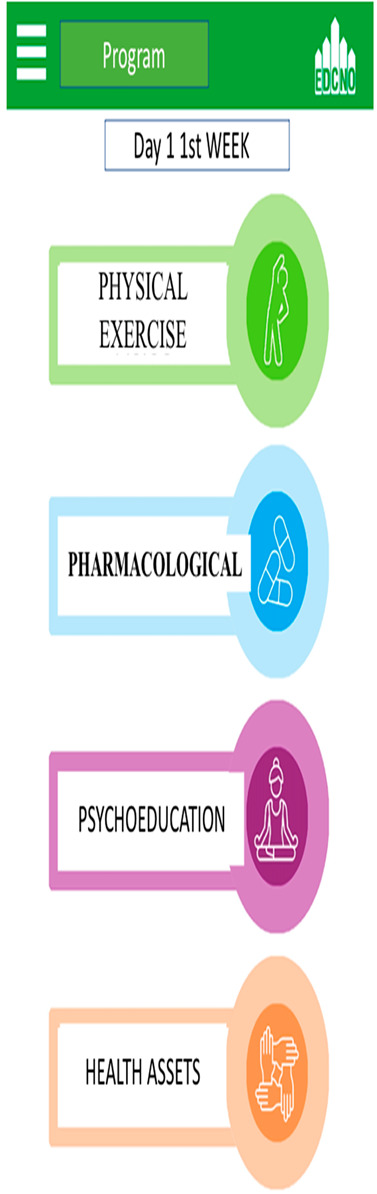	The activities in the exercise section try to help you understand tools and resources that will help you acquire habits and learn about alternatives to improve physical, mental, and emotional well-being. The exercises are oriented in such a way that they can be followed and adapted individually, facilitating the day-to-day process, acceptance and well-being. The proposed exercises and activities are empowerment exercises, stretching practice, relaxation, walks or daily low-intensity activities.
	The activities in the pharmacological section try to help you understand medications that improve pain intensity, know the most common side effects and warning signs that each medicine can cause, the characteristics of each pain-related medication, as well as the drugs that are more suited to your current state, depending on the pain scale.
	The activities in this section are about the relationship between the experience of pain and psychological suffering. The application intervenes in these aspects through exercises based on acceptance and commitment therapy and mindfulness. These therapeutic tools have been effective in achieving two main objectives, on the one hand, to promote greater acceptance of the experience, reducing the aversive component associated with pain by helping the individual to recognise and observe dispassionately both pain and thoughts as well as emotions that may arise. On the other hand, another group of activities raises awareness of the person’s values and the setup of an activity plan for the recovery of a significant vital project.
	The activities in the assets section seek to improve self-esteem and health, helping to identify resources in the environment with which you can more easily cope with situations of vulnerability and stress. These activities will help you feel better.

## Data Availability

The data supporting reported results can be found in the electronic notebook of the Data Entry Manager of IMIBIC. The statistician, principal investigator, and study collaborators who evaluated the results did not participate in patient recruitment.
